# Functions of Coronavirus Accessory Proteins: Overview of the State of the Art

**DOI:** 10.3390/v13061139

**Published:** 2021-06-13

**Authors:** Puxian Fang, Liurong Fang, Huichang Zhang, Sijin Xia, Shaobo Xiao

**Affiliations:** 1 State Key Laboratory of Agricultural Microbiology, College of Veterinary Medicine, Huazhong Agricultural University, Wuhan 430070, China; pxfang1990@163.com (P.F.); fanglr@mail.hzau.edu.cn (L.F.); zhanghuic74@126.com (H.Z.); sjx19940618@163.com (S.X.); 2 Key Laboratory of Preventive Veterinary Medicine in Hubei Province, the Cooperative Innovation Center for Sustainable Pig Production, Wuhan 430070, China; 3 Laboratory of Animal Virology, College of Veterinary Medicine, Huazhong Agricultural University, Wuhan 430070, China

**Keywords:** coronavirus, accessory protein, innate immunity, infection, pathogenesis

## Abstract

Coronavirus accessory proteins are a unique set of proteins whose genes are interspersed among or within the genes encoding structural proteins. Different coronavirus genera, or even different species within the same coronavirus genus, encode varying amounts of accessory proteins, leading to genus- or species-specificity. Though accessory proteins are dispensable for the replication of coronavirus in vitro, they play important roles in regulating innate immunity, viral proliferation, and pathogenicity. The function of accessory proteins on virus infection and pathogenesis is an area of particular interest. In this review, we summarize the current knowledge on accessory proteins of several representative coronaviruses that infect humans or animals, including the emerging severe acute respiratory syndrome coronavirus 2 (SARS-CoV-2), with an emphasis on their roles in interaction between virus and host, mainly involving stress response, innate immunity, autophagy, and apoptosis. The cross-talking among these pathways is also discussed.

## 1. Introduction

Coronavirus (CoV) infection usually causes mild respiratory symptoms, gastroenteritis, and hepatitis in humans and animals, but in some cases, it leads to life-threatening disease. For example, SARS-CoV and Middle East respiratory syndrome (MERS-CoV) infections lead to severe respiratory syndrome in humans [1,2], and porcine enteric diarrhea virus (PEDV) causes serious symptoms in animals [3,4]. Recently, as a newly emerged highly pathogenic agent of respiratory disease, SARS-CoV-2, has caused more than a mil-lion cases of infection around the world from 2019 to 2020 and led to a high fatality rate, thereby having a significant impact globally [5,6]. Although a great deal of research had previously focused on SARS-CoV and MERS-CoV, when the SARS-CoV-2 outbreak occurred, there were no available drugs or effective vaccines to prevent or treat the disease. These highly pathogenic CoVs pose an enormous threat to human and animals health and are now receiving extensive attention from researchers.

Coronaviruses belong to the subfamily *Coronavirinae* in the family *Coronaviridae* of the order *Nidovirales* and can be classified into four genera, including *Alphacoronavirus* (α-CoV), *Betacoronavirus* (β-CoV), *Gammacoronavirus* (γ-CoV), and *Deltacoronavirus* (δ-CoV) [7,8], which possess single-stranded and positive-sense RNA genomes. The α-CoVs and β-CoVs only infect mammals, including human CoVs (HCoV-229E, HCoV-OC43, SARS-CoVs, and MERS-CoV) and animal CoVs (mouse hepatitis virus (MHV), PEDV, feline infectious peritonitis virus (FIPV), porcine transmissible gastroenteritis virus (TGEV), swine acute diarrhea syndrome coronavirus (SADS-CoV)) [9,10,11]. These human CoVs cause infections of the respiratory tract, resulting in the common cold, severe pneumonia, acute respiratory distress syndrome (ARDS), and even death [12]. As economically important pathogens, PEDV, TGEV, and SADS-CoV pose a heavy disease burden on livestock [3,13]. The γ-CoVs mainly infect birds, with the most well-studied γ-CoV being infectious bronchitis virus (IBV) [14]. δ-CoVs such as bulbul CoV (BuCoV) and porcine deltacoronavirus (PDCoV) can infect birds and mammals [7,15,16]. PDCoV has the smallest genome among all CoVs and is a unique δ-CoV in that it can be passaged in vitro. Previous studies have shown that calves [17], chickens [18], and turkeys [19] are also susceptible to PDCoV. A recent study reported that PDCoV was identified in plasma samples from three children with acute undifferentiated febrile illness in Haiti [20], suggesting that PDCoV has the ability to infect humans, producing a significant threat to human and animal health [21].

**Figure 1:**
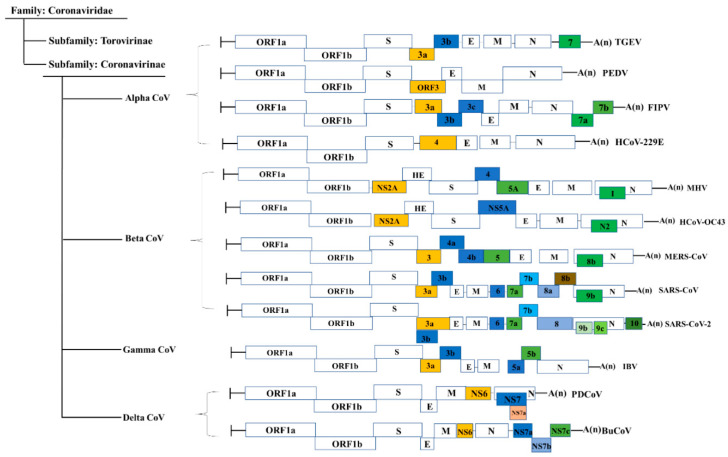
Genome organization of accessory genes from several representative coronaviruses. SARS-CoVs, severe acute respiratory syndrome coronaviruses; MERS-CoV, Middle Eastern respiratory syndrome coronavirus; HCoV-229E, human coronavirus 229E; HCoV-OC43, human coronavirus OC43; PEDV, porcine epidemic diarrhea virus; TGEV, transmissible gastroenteritis virus; MHV, mouse hepatitis virus; FIPV, feline infectious peritonitis virus; IBV, infectious bronchitis virus; BuCoV, bulbul coronavirus; PDCoV, porcine deltacoronavirus. Various accessory genes are shown as different colored boxes. ORFs 1a and 1b comprise the coronaviruses replicase genes. S, E, M, and N represent viral structural proteins. The figure is not drawn to scale.

Coronavirus genomic RNA consists of multiple open reading frames (ORFs) with an invariant gene order: 5′-replicase-S-E-M-N-3′, with a varying number of accessory protein genes scattered among or within the structural genes [7]. CoV accessory proteins are genus-specific and share no homology with known viral proteins, and each CoV contains a different number of accessory protein genes interspaced between or within the viral structural protein genes [22]. Figure 1 provides details on the organization of several representative CoV genomes. For example, FIPV, which is an α-CoV, possesses five accessory proteins, while another α-CoV, PEDV, has only one accessory protein, and the SARS-CoV of β-CoV has eight. SARS-CoV-2 possesses nine putative accessory proteins [23]. IBV, which is a γ-CoV, encodes four accessory proteins. As a representative of δ-CoVs, PDCoV has been experimentally proven to encode three accessory proteins [24,25]. Among all CoVs, SARS-CoV accessory proteins are the best characterized both biochemically and molecularly, although their functions still remain largely undefined [22]. On the basis of extensive reports, CoV accessory proteins are widely considered to be nonessential for in vitro viral replication but are vital for specific interactions between virus and host and are associated with protein incorporation into virus particles, apoptosis and inflammation induction, and the host’s antiviral response modulation, which may influence viral pathogenesis and disease outcomes [22,26,27]. Therefore, fully elucidating the functions of these accessory proteins is vital for our understanding of CoV pathogenesis and for developing effective antiviral drugs and vaccines.

In this review, we summarize current knowledge on the accessory proteins of several representative CoVs, including human and animal CoVs (SARS-CoVs, MERS-CoV, HCoV-229E, HCoV-OC43, MHV, FIPV, PEDV, TGEV, IBV, and PDCoV), with particular emphasis on the association of interactions between virus and host (stress response, innate immunity, autophagy, and apoptosis). We also propose the potential significance of these discoveries for further research.

## 2. Innate Immune Interferon (IFN) Responses

**Figure 2:**
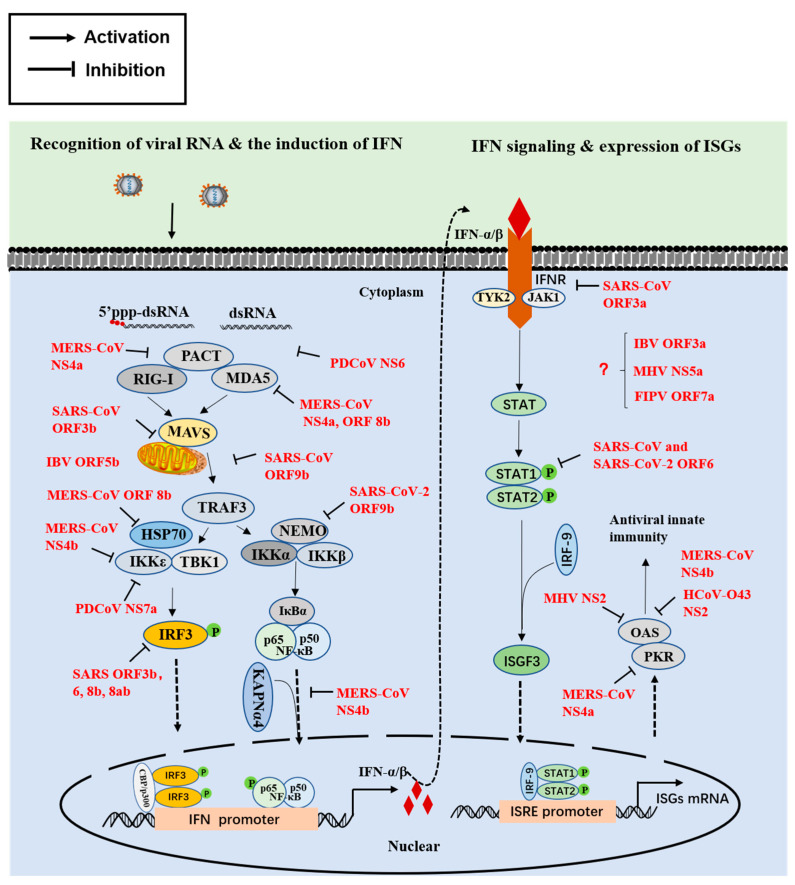
Regulation of innate immune interferon responses by coronavirus accessory proteins. Schematic diagram representing the induction and signaling transduction of type I interferon during coronavirus infection and the known modulatory mechanisms of accessory proteins. PRRs (RIG-I and MDA5) are activated to trigger a series of signaling pathway activations, such as IRF3 and NF-κB, for the induction of IFN. IFNs then bind to IFNAR and activate the JAK-STAT signaling pathway to trigger ISGs. Coronavirus accessory proteins regulating the pathway are shown in red. A question mark indicates the unknown IFN antagonistic mechanism. Abbreviations: dsRNA, double-stranded RNA; PACT, protein activator of protein kinase R; RIG-I, retinoic acid-inducible gene I; MDA5, melanoma differentiation-associated gene 5; MAVS, mitochondrial antiviral signaling protein; TRAF3, TNF receptor-associated factor 3; HSP70, heat shock protein 70; TBK1, TANK-binding kinase 1; IKKε, inhibitor of κB kinase ε; IRF3, interferon regulatory factor 3; NEMO, NF-κB essential modulator; IKKα, inhibitor of κB kinase α; IKKβ, inhibitor of κB kinase β; IκBα, inhibitor of nuclear factor kappa-B; NF-κB, nuclear factor kappa-B; KAPN-α4, karyopherin-α4; TYK2, tyrosine kinase 2; JAK1, Janus kinase 1; STAT1/2, signal transducer and activator of transcription 1/2; IRF9, interferon regulatory factor 9; ISGF3, IFN-stimulated gene factor 3; OAS, oligoadenylate synthetase; PKR, protein kinase R; ISRE, IFN-stimulated response element; P, phosphate.

The immune response mediated by type I IFN (IFN-I) is the first line of antiviral defense. Melanoma differentiation gene 5 (MDA5) is the most important sensor for the recognition of CoV replicative intermediates (pathogen-associated molecular patterns, PAMPs) [28]. Upon binding to PAMPs, MDA5 and RIG-I are activated, resulting in the aggregation of mitochondrial signaling adapter (MAVS) and TANK binding kinase 1 (TBK1)/I kappaB kinase (IKKε) activation, and subsequently, phosphorylation and dimerization of IRF3 and nuclear factor κB (NF-κB), followed by their entry into the nucleus and IFN-I production [29,30]. The IFN-I binds to IFN-α/β receptors, inducing the JAK/STAT signaling pathway activation and numerous of IFN-stimulated genes (ISGs) production. Expression of ISG induced an amplification of response in the first two levels by different positive feedback loops, as evidenced by the induction of IFN-α. Finally, some ISGs-encoded antiviral activities collectively induce the establishment of an antiviral state and antagonism action against a broad range of RNA viruses [31]. A feeble IFN-I response appears to be a hallmark of infections from CoVs, including human CoVs, SARS-CoV, MERS-CoV [32], and animal CoVs, PEDV, MHV, and PDCoV [33,34]. Recent studies indicated that SARS-CoV-2 infection induced a disorganized immune response, featured by a poor and delayed IFN-I response and exacerbated proinflammatory cytokines production, leading to serious illness [35]. Taken together, similar to many other viruses, CoVs have evolved effective strategies for escaping the IFN system at three levels, including inhibiting IFN induction, blocking IFN signal transduction, and opposing the action of antiviral proteins by various mechanisms, therefore ensuring their survival [36,37]. The identification and characterization of viral proteins that antagonize the IFN response have been continually reported. Among them, CoV accessory proteins play crucial roles in impairing the IFN responses at different critical points, ranging from the recognition of PAMPs to the action of antiviral proteins in these pathways. A detailed description of the IFN antagonistic mechanism of CoV accessory proteins is presented below, and a corresponding schematic diagram is shown in Figure 2. 

### 2.1. The First Step Involves IFN Induction

The recognition of PAMPs by cellular RNA sensor RIG-I/MDA5 is the initial step, which was targeted by various accessory proteins via different strategies, such as sequestration of dsRNA recognition by MDA5 [38] and inhibition of interaction between double-stranded RNA-binding protein with RIG-I or MDA5 [39] by MERS-CoVNS4a protein, attenuation of dsRNA binding to RIG-I/MDA5 by PDCoV NS6 protein [40], and an unknown mechanism for MERS-CoV ORF8b [41]. It appears that RIG-I and MDA5 are a key target of CoVs, as well as numerous other viruses [42,43]. As key adaptor proteins, MAVS and TBK1/IKKε play a crucial role in the host’s innate immune response modulation, and they are also targeting for many CoVs to antagonize IFN production. SARS-CoV ORF3b may target MAVS to inhibit IFN induction because it is translocated to mitochondria when overexpressed in Vero cells [44,45]. SARS-CoV ORF9b acts on mitochondria and targets the MAVS signalosome (MAVS, TRAF3, and TRAF6) via seizing poly(C)-binding protein 2 (PCBP2) and the HECT domain E3 ligase AIP4 to induce its degradation, limiting the production of IFN production [46]. Like SARS-CoV, SARS-CoV-2 ORF9b interacts with MAVS by associating with Tom70, implying a conserved IFN antagonistic mechanism [47]. A recent report indicated a novel IFN inhibitory mechanism by which SARS-CoV-2 ORF9b interrupts the K63-linked ubiquitination of NEMO to inhibit the RIG-I-MAVS signaling pathway, suggesting a variety of antagonistic actions of SRAS-CoV-2 ORF9b [48]. Both TBK1 and IKKε are effective for IFN induction and are often employed by viruses to disturb the IFN signaling response, such as via directly interacting with both IKKε and TBK1 as observed for MERS-CoV NS4b [49] or via competing specifically with IKKε for interaction with HSP70 as observed for MERS-CoV ORF8b [50]. Our recent study showed that PDCoV NS7a specifically interacts with IKKε but not with TBK1, interfering with IKKε binding to upstream TRAF3 and downstream IRF3 [51]. Multiple CoV accessory proteins hijacked IRF3 and NF-κB for IFN production to facilitate viral replication, such as SARS-CoV ORF3b and ORF6. It is speculated that ORF3b protein may interact with nuclear transcription factors necessary for the IFN induction and that ORF6 may disrupt ER/Golgi transport necessary for IFN induction [44]. A recent study indicated that SARS-CoV ORF8b and ORF8ab suppress IFN production via rapid degradation of IRF3 mediated in a ubiquitin-dependent manner [52]. Furthermore, MERS-CoV NS4b showed a strong preference for binding to karyopherin-α4 (KPNAα4), outcompeting the NF-κB p65 subunit for KPNAα4 binding, and inhibiting the NF-κB-mediated IFN response during infection [53]. These data suggest that various CoV accessory proteins can effectively target key molecules in the IFN pathway mediated by RLRs to escape the host antiviral immune response, suggesting that IFN inhibition by CoV accessory proteins is a common feature. 

Several accessory proteins are potential IFN antagonists, such as HCoV-OC43 NS2a and NS5a as well as MERS-CoV ORF5, but the molecular mechanisms involved need to be further investigated [54,55]. SARS-CoV-2 is sensitive to type I IFN pretreatment. However, SARS-CoV-2 also encodes multiple proteins to counteract IFN production, including non-structural proteins (nsp1, nsp3, nsp12, nsp13, nsp14), two structural proteins (M, N), and three accessory proteins (ORF3b, ORF6, ORF8) [56]. Among them, ORF3b suppresses IFN induction more efficiently than its SARS-CoV orthologue, and a natural variant with a longer ORF3b that displayed stronger inhibitory activity was identified through screening 15,000 SARS-CoV-2 strains [57,58]. SARS-CoV-2 ORF8 can form unique large-scale assemblies that are not possible for SARS-CoV, as determined by a crystal structure assay, potentially contributing to mediation of immune suppression and evasion activities [59]. This immune suppression of the IFN-I signaling pathway was further confirmed through an overexpression experiment [56]. Furthermore, SARS-CoV-2 ORF8 could downregulate MHC-I expression to mediate immune evasion. These findings suggest that SARS-CoV-2 ORF8 escapes the host immune system using a variety of strategies, unlike the SARS-CoV orthologue. Although SARS-CoV-2 has high nucleotide homology to SARS-CoV, SARS-CoV-2 is more sensitive to IFN treatment, suggesting their distinguishing regulation of IFN signaling. Further identification and characterization of the SARS-CoV-2-encoded IFN antagonist will facilitate a greater understanding of the relationship of SARS-CoV-2 with the IFN signaling pathway, as well as this potential pathogenic mechanism.

### 2.2. The Second Step Involves Signal Transduction

SARS-CoV encodes multiple accessory proteins to impede IFN signaling transduction via different strategies, such as via reducing IFNAR expression by SARS-CoV ORF3a [60], binding to karyopherin-α2 to prevent nuclear translocation of STAT1 by SARS-CoV ORF6 [61], and another so far poorly characterized mechanism by SARS-CoV ORF3b [44]. Cheng and colleagues found that SARS-CoV ORF6 interacted with host N-Myc (and STAT) interactor (Nmi) protein and induced its degradation, blocking the host antiviral immune response via impairing Nmi-mediated IFN-stimulated response element (ISRE) activation [62]. SARS-CoV-2 ORF6 displayed a similar distribution and comparable ability to impair STAT1 nuclear translocation with its SARS-CoV orthologue, and it was further demonstrated that several amino acids at the end of the ORF6 C-terminus were indispensable for the function of ORF6 at inhibiting STAT1 activation [57]. Miorin and coworkers then provided direct evidence that SARS-CoV-2 ORF6 blocks STAT nuclear translocation via interacting with NUP98 and RAE1 [63]. Additionally, several CoV accessory proteins were proven to function as inhibitors of the IFN signaling response during virus infection. FIPV ORF7a protein can protect the virus from an IFN-mediated antiviral state in the context of ORF3 protein expression, suggesting a synergistic effect between ORF7a and ORF3 [64]. MHV NS5a and its homologues from β-CoVs antagonize the antiviral action of IFN during virus infection [65]. IBV ORF3a is also associated with resistance to IFN treatment because its absence leads to rendering IBV less resistant to IFN [66]. However, the antagonistic mechanism requires further study. Overexpression experiments indicated the strong inhibitory effect on ISRE promoter activity by numerous CoV accessory proteins, such as HCoV-OC43 NS2a and NS5a, MERS-CoV NS4a and NS4b [54], as well as three PDCoV-encoded accessory proteins (NS6, NS7, NS7a); however, this inhibitory mechanism needs to be verified during virus infection. Taken together, these data suggest that accessory proteins from various CoV genera extensively participate in the host innate IFN response, as confirmed in a recent report [67].

### 2.3. The Final Step Involves the Activity of Antiviral Proteins

On the basis of the direct antiviral effects of ISGs, the identification and characterization of accessory proteins inhibiting ISG activity may facilitate the discovery of antiviral targets. To date, several accessory proteins have been reported to directly target ISGs to inhibit antiviral activity. TGEV protein 7 was shown to limit RNase L activation because its deletion led to increased RNA degradation that was involved in the RNase L system [68], but the molecular mechanism involved was not further explored. MERS-CoV NS4a suppresses PKR-dependent antiviral stress responses via sequestering dsRNA, facilitating viral replication [69,70]. MHV NS2 acts as a 2’,5’-phosphodiesterase that cleaves 2’,5’-oligoadenylates, blocking the IFN inducible 2’,5’-oligoadenylate synthetase (OAS)-RNase L pathway [71]. Similar functional viral proteins include the homologues HCoV-O43 NS2 [72] and MERS-CoV NS4b [73]. More inhibitory mechanisms await further exploration.

## 3. Innate Proinflammatory Immune Response

**Figure 3:**
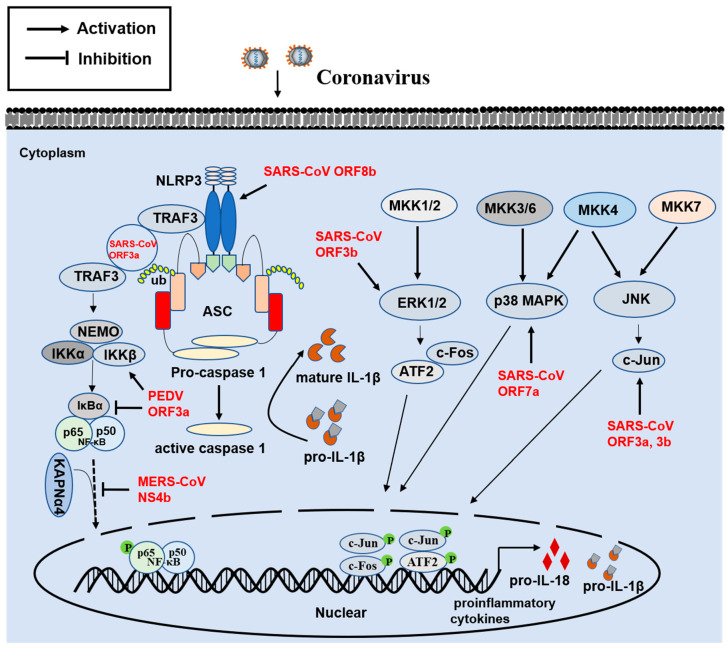
Modulation of an innate immune proinflammation response by coronavirus accessory proteins. Schematic diagram showing the activation and modulation of NLRP3 inflammasome and the MAPK signaling pathway by accessory proteins. Accessory proteins modulating the pathway are shown in red. Abbreviations: NLRP3, NLR family pyrin domain containing 3; TRAF3, TNF receptor-associated factor 3; ASC, apoptosis-associated speck-like protein containing a caspase recruitment domain; NEMO, NF-κB essential modulator; IKKα, inhibitor of κB kinase α; IKKβ, inhibitor of κB kinase β; KAPN-α4, karyopherin-α4; MAPK, mitogen activated protein kinases; MKK, MAP kinase kinases; ERK1/2, extracellular signal-regulated kinase 1/2; JNK, c-Jun N-terminal kinases; c-Fos, cellular FBJ murine osteosarcoma; ATF2, activating transcription factor 2; P, phosphate.

The proinflammatory cytokine storm is a hallmark of infections caused by SARS-CoVs and MERS-CoV. Using SARS-CoV-2 as an example, SARS-CoV-2 infection can display a series of clinical symptoms, ranging from asymptomatic to severe disease. Severe disease symptoms are featured by pneumonia, progression to ARDS, shock and multiorgan dysfunction, and even death [74]. Severe COVID-19 is involved in the inflammatory cytokine storm and inflammatory events, such as increased proinflammatory cytokine levels, including tumor necrosis factor-α (TNF-α), interleukin 1 (IL-1), interferon-γ inducible protein 10 (IP-10), and IL-6 [75]. A number of studies have suggested that SARS-CoV-2 triggers IL-1β and IL-18 expression by activating the Nod-like receptor family and pyrin domain-containing 3 (NLRP3) inflammasome, leading to severe lung injury and ARDS [76]. As a central inflammatory multimeric complex, inflammasome mainly consists of pro-caspase-1, apoptosis-associated speck-like protein containing a caspase recruitment domain (ASC), and NLRP3 protein, which can be activated by PAMPs, damage-associated molecular pattern molecules, and reactive oxygen species. Accumulating evidence shows that NLRP3 specifically interacts with the pyrin domain of ASC, followed by the recognition of stimulus-mediated oligomerization and the recruitment of pro-caspase-1 by ASC [77]. Upon activation, caspase-1 can trigger the cleavage of pro-IL-1β and pro-IL-18 into mature IL-1β and IL-18, respectively, leading to an inflammatory response (Figure 3). Several studies have indicated that E, ORF3a, and ORF8b of SARS-CoV function as NLRP3 agonists. SARS-CoV ORF3a specifically interacts with TRAF3 to induce the transcription of pro-IL-1β gene via activating the NF-κB p50 subunit and subsequent caspase 1 and IL-1β maturation through promoting ASC ubiquitination [78]. SARS-CoV ORF8b directly interacts with the leucine-rich repeat domain of NLRP3 and localizes in cytosolic dot-like structures with NLRP3 and ASC, inducing NLRP3 inflammasome activation and subsequently increasing IL-1β and IL-18 levels in macrophages and potentially in lung epithelial cells [79]. Due to the conserved protein sequences, these homologous proteins in SARS-CoV-2 likely also play vital roles in inflammatory pathogenesis. Considering the potential function of NLRP3 in SARS-CoVs infection-mediated inflammatory responses, blocking inflammatory cytokines and the NLRP3 inflammasome may be a promising strategy for limiting the effects of COVID-19. Indeed, increasing evidence shows that some drugs against NLRP3 inflammasome, such as Glyburide, MCC950, and OLT1177, could reduce inflammatory responses, alleviating clinical symptoms in patients [80]. Taken together, these findings indicate that the NLRP3 inflammasome may be a therapeutic target by which to limit pathological and clinical manifestations. 

Except for the abovementioned mechanisms, other CoV accessory proteins associated with the modulation of proinflammatory cytokine expression have been reported via various mechanisms (Figure 3). SARS-CoV ORF3a can promote the activities of NF-κB and c-Jun N-terminal protein kinase (JNK) and can increase TNF-α, IL-8, and CCL5 expression in murine macrophages and lung cell lines [81,82]. SARS-CoV ORF7a also induces p38 mitogen-activated protein kinase (p38 MAPK) and NF-κB activation, rather than JNK, resulting in the increased expression of IL-8 and CCL5 [81,83]. SARS-CoV ORF3b induces the transcription of AP-1 by the activation of the JNK and ERK pathways, leading to the upregulation of CCL2 in a human hepatoma cell line [84,85]. As a result of the high homology between SARS-CoV-2 and SARS-CoV, the corresponding accessory proteins of SARS-CoV-2 might display similar roles, although this needs to be further investigated. Other animal CoVs can also induce significantly increased proinflammatory cytokines expression during viral infection, such as TGEV [86], PEDV [3], and PDCoV [87]. For PEDV, ORF3 protein interacts with the IκB kinase β (IKBKB), promoting NF-κB promoter activity mediated by IKBKB [88]. However, another recent study showed that PEDV ORF3 protein disrupts the phosphorylation and degradation of IκBα, blocking phosphorylation and the nuclear translocation of p65 and resulting in downregulation of IL-6 and IL-8 production [89]. The author indicated that various stages of viral infection might be a cause of contradictory results. Furthermore, TGEV protein 7 showed an inhibitory effect on the expression of proinflammatory cytokines [90], but the molecular mechanism was not further investigated. The negative regulation of proinflammatory cytokines was also reported in MERS-CoV NS4b, which competes with the NF-κB-p65 subunit for binding to KPNAα4 and leads to the attenuation of NF-κB-mediated proinflammatory cytokine expression [53]. Taken together, the function of accessory proteins in the modulation of inflammatory cytokine expression during virus infection is a double-edged process because they may have pro- or anti-inflammatory roles. Investigating the precise mechanism of proinflammatory cytokines induction by highly pathogenic viruses might provide clues in terms of therapeutic interventions.

## 4. Involvement of ER Stress, Apoptosis, and Autophagy

**Figure 4:**
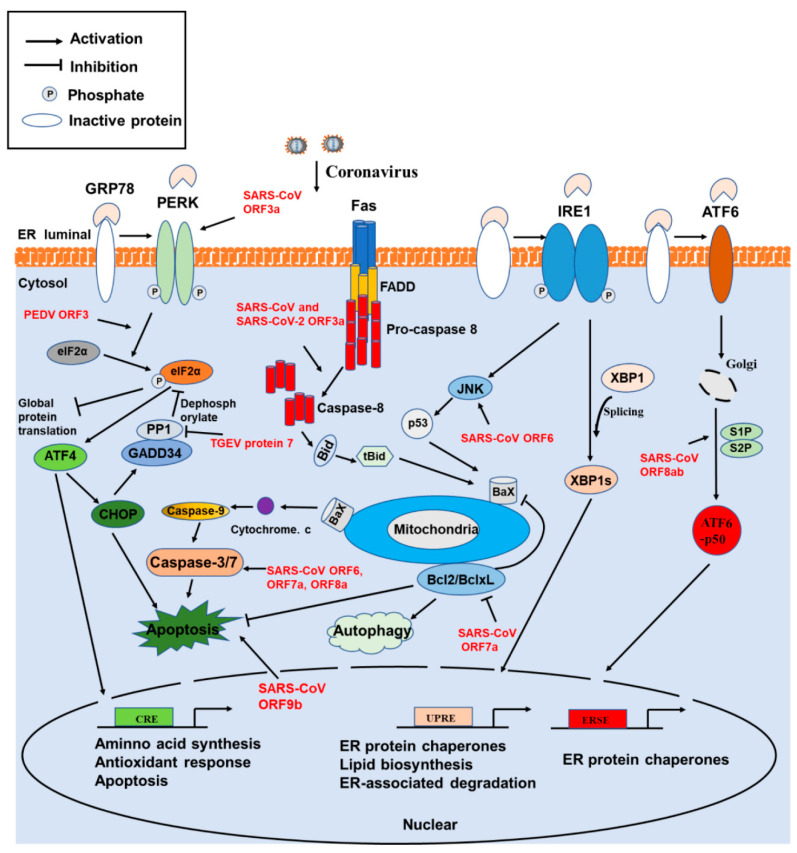
Modulation of unfolded protein responses, apoptosis, and autophagy by coronavirus accessory proteins. Schematic diagram showing the three arms of UPR signaling pathways and the correlated apoptosis and autophagy pathways regulated by accessory proteins. Accessory proteins modulating the pathway are shown in red. Abbreviations: PERK, PKR-like ER protein kinase; IRE1, inositol-requiring enzyme 1; ATF6, activating transcription factor 6; GRP78, glucose-regulated protein, 78 kDa; eIF2α, eukaryotic initiation factor 2 subunit α; ATF4, activating transcription factor 4; CRE, cAMP response element; CHOP, C/EBP-homologous protein; PP1, protein phosphatase 1; GADD34, growth arrest and DNA damage inducible 34; UPRE, unfolded protein response element; ERSE, ER stress response element; XBP, X-box-binding protein; JNK, c-Jun N-terminal kinases; Bcl-2, B-cell lymphoma 2/XL; Bcl-xL, Bcl-2-like protein 1; S1/2P, site-1/2 protease; FADD, Fas associated via death domain; BaX, Bcl2-associated X; Bid, BH3-interacting domain.

The ER is the major organelle necessary for protein synthesis, folding, and trafficking. When the ER is stressed and overwhelmed due to the substantial accumulation of unfolded proteins, it can cause an ER stress response. To cope with ER stress and maintain protein homeostasis, host cells initiate the unfolded protein response (UPR), which consists of three known interrelated signaling branches: protein kinase RNA-activated (PKR)-like ER protein kinase (PERK), activating transcription factor 6 (ATF6), and inositol-requiring enzyme 1 (IRE1) (Figure 4). The UPR pathway is intricate, and its activation modulates a wide variety of signaling pathways, such as MAPK activation, autophagy, apoptosis, and the innate immune response [91]. ER stress and UPR activation are major contributors to the pathogenesis of several diseases, such as inflammatory disorders and viral infections [92]. Importantly, chronic UPR activation has been described in many diseases, such as diabetes, cancer, and neurodegeneration. CoV replication occurs in the cytoplasm and is closely related to the ER. During CoV infection, excessive production of viral proteins and the formation of double-membrane vesicles and viral envelops often result in ER stress. Thus, the induction of ER stress is likely a common phenomenon in CoV-infected cells. Multiple CoV infections, including those caused by SARS-CoV [93], MHV [94], TGEV [95], IBV [96], SARS-CoV-2 [97,98], and PDCoV (unpublished data), have been confirmed to induce obvious ER stress. Due to prolonged periods of stress, the exasperation of the UPR could lead to activation of apoptotic pathways and cell death. Thus, CoV-induced apoptosis is also a common phenomenon, occurring in infections caused by TGEV [99], PEDV [100,101], FIPV [102], HCoV-OC43 [103], HCoV-229E [104], SARS-CoV [105], MERS-CoV [106,107], MHV [108,109], IBV [110], and PDCoV [111,112]. Although the induction of autophagy was also investigated, in particular in PEDV, MHV, IBV, and PDCoV [113,114], the detailed molecular mechanism is less clearly defined than the above two stress responses. However, studies on the interaction between SARS-CoV-2 and autophagy have received widespread attention, and many researchers have focused on this topic and on targeting the autophagy pathway as a strategy for treating COVID-19 [115,116]. For example, multiple autophagy modulators could block the SARS-CoV-2 cytopathic effect [117]. Certainly, more work is needed in the future before achieving an effective and safe drug against the autophagy pathway.

Some studies have identified and characterized multiple accessory proteins involved in the modulation of ER stress, apoptosis, and autophagy. For example, SARS-CoV ORF8ab is an ER membrane-associated protein and induces the activation of ATF6 pathway via interacting with ATF6 [118]. SARS-CoV ORF8b and ORF6 also trigger the activation of ER stress [79,119]. A previous study demonstrated that TGEV protein 7 bound to protein phosphatase 1 catalytic subunit (PP1c), leading to an increase in eIF2α phosphorylation [68]. It is possible for TGEV protein 7 to modulate the PERK-eIF2α pathway, but further investigations are needed. Regarding apoptosis, multiple accessory proteins of SARS-CoV are involved in this process via different molecular mechanisms when overexpressed in cells, such as ORF3a, ORF3b, ORF6, ORF7a, ORF8a, and ORF9b. Among them, ORF3a has been confirmed to trigger apoptosis by activating the p38 MAPK of the mitochondrial death pathway [120] and to drive multimodal necrotic cell death [121]. ORF6 triggers caspase-3 mediated and ER stress-dependent apoptosis pathways [119]. Tan and colleagues demonstrated that ORF7a protein triggers apoptosis by interacting with Bcl-X_L_, interfering directly with its pro-survival function, as well as by a caspase-dependent manner [122,123]. ORF8a protein induces caspase 3 activity and cellular apoptosis through a mitochondria-dependent pathway and promotes viral infection [124]. SARS-CoV ORF9b protein can induce apoptosis due to its retention in the nucleus [125]. Recently, ORF3a, encoded by SARS-CoV-2, was identified as a strong inducer of caspase-dependent apoptosis in multiple cells [126]. By contrast, a report indicated that PEDV ORF3 inhibits the apoptosis of infected cells to facilitate viral proliferation [127].

During coronavirus infection, widespread crosstalk occurs among multiple host cell signaling pathways. The ERK and IRE1 participate in the modulation of autophagy induction in the context of IBV infection [128]. The activated IRE1 antagonizes IBV-induced apoptosis to facilitate cell survival during viral infection [129]. Furthermore, it was demonstrated that SARS-CoV ORF3a protein induces ER stress and downregulates IFN-I receptor expression, leading to the inhibition of the IFN response [60]. PEDV ORF3 activates ER stress to facilitate autophagy [130]. The identification and characterization of more CoV accessory proteins that participate in cell signaling modulation await further investigation. Generally speaking, ER stress, apoptosis, and autophagy, which are involved in cellular homeostasis and immune responses to viral infections, have dual functionality in virus infections because they have both pro- and anti-viral roles. Characterization of the precise crosstalk among ER stress, apoptosis, and autophagy in CoV-infected cells would deepen our understanding of interactions between virus and host and help to discover potential antiviral targets.

## 5. The Action Mediating the Ion Channel Activity of Viroporins

Viroporins are virus-encoded proteins that mediate ion channel (IC) activity, playing crucial roles in virus infection and pathogenesis. Viroporins are characterized by their small size, hydrophobicity, and their ability to permeabilize membranes by oligomerization [131]. These viroporins consist of many multifunctional proteins from various viral families that are predominately concentrated in RNA viruses, including influenza A virus (IAV), hepatitis C virus (HCV), respiratory syncytial virus (RSV), picornaviruses, and CoVs (e.g., SARS-CoVs and MERS-CoV) [132,133,134,135,136,137]. Many CoVs encode two or more viroporins, such as E protein, with extra viroporins encoded by accessory genes (Table 1). Several studies have confirmed that the E proteins are harbored by a range of CoVs, including HCoV-229E, SARS-CoVs, MERS-CoV, MHV, and IBV, and that they display vital functions as viroporins involved in viral infection and pathogenesis [137,138,139].

Generally speaking, the removal of E protein is deleterious to CoVs, leading to the blockage of virus trafficking, impairment of virion assembly and maturation, and an attenuated phenotype [145,146,147]. CoV accessory proteins, with the exception of ORF3 protein between S and E, are generally small proteins. SARS-CoV ORF3a and ORF8a have been confirmed to function as an ion channel and to promote virus release [140,141,148,149], as well as PEDV ORF3 [142]. HCoV-229E ORF4a is functionally analogous to SARS-CoV ORF3a, acting as a viroporin to modulate virus proliferation [143]. HCoV-OC43 ns12.9 functions as a viroporin and is closely associated with virion morphogenesis and pathogenesis [144]. Interestingly, SARS-CoV ORF3a, HCoV-229E ORF4a, and HCoV-NL63 ORF3 could restore the growth of a ns12.9 deletion mutant of HCoV-OC43, suggesting that they share a conserved viroporin function [144]. 

On the basis of previous studies, it was found that these viroporins share similar localization, mainly at the ER/Golgi intermediate compartment (ERGIC) or Golgi compartment, which are the sites of CoV assembly and packaging. Partial viroporins are incorporated into virus particles, such as E and ORF3a, which may explain the crucial role that many viroporins play in viral infection. Viroporins, as well as cellular IC proteins, frequently contain PDZ domains and PDZ-binding motifs (PBMs) that are generally located at their C-terminus, contributing to their wide interaction with host proteins. Previous studies have confirmed that SARS-CoV ORF3a and E possess a class II PBM [150]. Similar motifs are also present in proteins E and 5 encoded by MERS-CoV. Therein, E protein is verified to be a viroporin, and protein 5 is expected to possess a similar function [136]. Additional studies are needed to demonstrate which accessory proteins function as viroporins in γ-CoV and δ-CoV. Our previous study demonstrated that PDCoV regulates the calcium influx to facilitate viral infection and that an interaction of PDCoV NS6 with STIM1 necessary for the modulation of SOCE occurs [151]. Interestingly, STIM1 acts a calcium sensor in the ER, and NS6 is primarily located at the ER/ERGIC in the context of virus infection, allowing for its interaction with STIM1 and the regulation of Ca2^+^ homeostasis [25]. Many reports have indicated that some viroporins, such as rotavirus nsp4, IAV M2, HCV p7, and picornavirus P2B protein, enhance the passage of ions and small molecules across membranes to promote viral proliferation [152,153,154,155]. Therefore, it is interesting to investigate whether NS6 is also a viroporin, and this work is currently underway in our laboratory. The identification and characterization of SARS-CoV-2 accessory proteins as viroporins have not yet been reported, but it is likely that SARS-CoV-2 encodes multiple viroporins because of its high homology to SARS-CoV. The vital role of viroporins in facilitating viral infection and pathogenicity has attracted increasing interest from many researchers due to their potential as drug targets [156]. The identification of some compounds that disturb the IC activity of viroporins and impair virus production has been reported [134,157,158,159]. Moreover, due to their association with viral pathogenesis, viroporin-defective viruses may be explored as live attenuated vaccines [160]. Therefore, the identification and characterization of viroporins facilitates the development of antiviral strategies and may potentially lead to effective vaccines.

## 6. Involvement of Virulence

To date, CoV reverse genetic systems have been extensively applied to investigate the functions of accessory proteins in vivo, demonstrating that accessory proteins are associated with viral virulence and play important roles in infection and pathogenesis. Thereby, their disruption may serve as a powerful tool for developing effective live attenuated vaccines. Mutant FIPVs with deleted ORF3abc or ORF7ab proliferated in vitro but displayed an attenuated phenotype in cats. Importantly, these mutant viruses provided effective protection for cats against a lethal challenge, suggesting their potential as a modified live FIPV vaccine [161]. Dedeurwaerder and colleagues found that ORF7ab deletion could not maintain viral replication, suggesting a vital role of ORF7 for FIPV replication in vitro and in vivo [162]. This suggested that the role of FIPV accessory proteins may be cell-specific. Similar to a report by Haijema and coworkers, the deletion of TGEV ORF3a and ORF3b (homologues of FIPV ORF3a and 3c) led to strong virulence attenuation [163]. Although ORF6 is not essential for the replication of SARS-CoV, it can significantly enhance the virulence of an attenuated murine CoV, suggesting its role in virulence [164]. The absence of four accessory genes (ORF3, -4a, -4b, and -5(dORF3-5)) encoded by MERS-CoV induced significant attenuation in a dORF3-5 mutant in vitro and in vivo [165]. For MHV, the deletion of multiple accessory genes in combination resulted in the production of attenuated recombinant viruses (MHV-Δ2aHE, MHV-Δ45a, MHV-Δ2aHE45a) compared with the wild-type virus. Among these recombinant viruses, MHV-Δ2aHE45a was more severely attenuated than the other deletion viruses [166]. The deletion of multiple accessory genes of IBV strain H52 BI generated an attenuated phenotype for the recombinant viruses (rIBV-Δ3a, rIBV-Δ3b, rIBV-Δ3ab, rIBV-Δ5ab, and rIBV-Δ3ab5ab) in 1-day-old SPF chickens and showed the ability to induce protection for chickens [167]. Zhao and colleagues further evaluated the effects of ORF3a and ORF3b on the pathogenicity of an IBV QX-like strain and found that their absence also led to attenuated pathogenicity in chickens [168]. These phenotypes are consistent with the ability of the encoded proteins to antagonize the IFN response [66,169]. The deletion of NS6 in PDCoV led to a substantial reduction of viral titer in vitro and in vivo, and piglets infected with the NS6 deletion mutant virus did not show any clinical scores or pathological injuries, suggesting that NS6 is a vital virulence factor [170]. With the exception of NS6, PDCoV encodes another two accessory proteins, NS7 and NS7a; whether deletion of two or three of these proteins is a more suitable option for developing attenuated live vaccines awaits further investigation. These issues are currently being addressed in our laboratory. SARS-CoV-2 is the etiologic agent of COVID-19, which has caused enormous loss of human life and has attracted unprecedented attention from researchers. Thus, the reverse genetic system of SARS-CoV-2 was rapidly and successfully established via multiple strategies, including the BAC-based system and a T7-based system [171,172], which were then used to explore the biology of the viral infection process, to evaluate the efficacy of antivirals or neutralizing antibodies, and to identify viral receptors via constructing recombinant viruses expressing reporter genes (mCherry, GFP, and Nluc) through the replacement of accessory genes [173,174]. A recent study identified ORF3a and ORF6 of SARS-CoV-2 as the major contributors of viral pathogenesis through a reverse genetics system to individually remove viral 3a, 6, 7a, 7b, and 8 ORF proteins [175]. However, the construction of recombinant SARS-CoV-2 with multiple accessory genes deletion is yet to be reported. Taken together, accessory proteins may function in combination with other accessory proteins encoded by the same CoV; hence, the simultaneous deletion of multiple accessory genes may be a promising strategy for the development of live attenuated vaccine.

## 7. Concluding Remarks and Future Prospects

Coronavirus accessory proteins are extensively involved in the host immune response and are often involved in virulence. Therefore, research on the functions of accessory proteins has become a hot spot in the field of CoV research in recent years. In this review, we summarized recent progress on the function of accessory proteins from several representative human and animal CoVs, with emphasis on crucial signaling pathways. Accessory proteins often function in combination, rather than individually. They not only coordinate the various processes of the viral replication cycle, but also modulate host immunity, including the stress response, autophagy, apoptosis, and innate immunity, providing an appropriate intracellular and extracellular environment for viral replication. However, the detailed molecular mechanisms used by accessory proteins remain largely unclear, especially in the context of infection. Currently, several key questions remain to be addressed: (1) Why does the number of accessory genes vary among CoVs from different genera, including α-CoV, β-CoV, γ-CoV, and δ-CoV, and even among the same CoV genus, and are the functions of the absent accessory proteins conducted by other proteins encoded by the same CoV? (2) Does an increase or decrease in the number of accessory genes from various CoVs contribute to the improved adaptability of CoVs to a new host or to cross-species transmission? (3) Since CoVs employ diverse translational strategies that are not yet fully understood, do CoVs encode more other unknown accessory proteins? (4) From where do these accessory genes derive and are they involved in broad host spectrums of CoVs, such as SARS-CoV-2 and PDCoV? (5) Which viral proteins interact with accessory proteins, and what is the biological significance of the interaction? (6) Do interactions occur among different accessory proteins from various CoVs with the same target organs, such as PDCoV, PEDV, and TGEV, resulting in more serious co-infections? The answers to these questions will deepen our understanding of the function of accessory proteins in CoVs and will facilitate the formulation of antiviral strategies and the development of effective vaccines.

With the advent of new bioinformatic tools and the availability of infectious clones and genome editing (CRISPR), new accessory proteins are constantly being identified [176,177,178,179]. Moreover, more signaling pathways and host factors targeted by accessory proteins are expected to be uncovered and characterized. Supplemented with the advances in molecular biology techniques, crystallization protocols, and NMR techniques, the biological structures of more accessory proteins will be elucidated, which will facilitate their molecular characterization and boost the rational design of specific antiviral inhibitors. In summary, future studies of accessory proteins from multifarious perspectives will facilitate a deeper understanding of the biology of these important viruses and will aid our ability to treat and prevent infections.

## Figures and Tables

**Table 1:** Accessory proteins of coronaviruses that function as ion channel proteins.

Coronavirus	Viral Channel	Aa Residues	TM	References
SARS-CoV	ORF3a	274	3	[140]
	ORF8a	39	1	[141]
PEDV	ORF3	225	4	[142]
HCoV-229E	ORF4a	133	3	[143]
HCoV-OC43	ns12.9	110	1	[144]

## Data Availability

The data presented in this study are available in the article.

## References

[1] Forni D., Cagliani R., Clerici M., Sironi M. (2017). Molecular Evolution of Human Coronavirus Genomes. Trends Microbiol..

[2] Coleman C., Frieman M.B., Pauck A., Lener B., Hoell M., Kaiser A., Kaufmann A.M., Zwerschke W., Jansen-Dürr P., Imperiale M.J. (2014). Coronaviruses: Important Emerging Human Pathogens. J. Virol..

[3] Jung K., Saif L.J., Wang Q. (2020). Porcine epidemic diarrhea virus (PEDV): An update on etiology, transmission, pathogenesis, and prevention and control. Virus Res..

[4] Bi J., Zeng S., Xiao S., Chen H., Fang L. (2012). Complete Genome Sequence of Porcine Epidemic Diarrhea Virus Strain AJ1102 Isolated from a Suckling Piglet with Acute Diarrhea in China. J. Virol..

[5] Li R., Pei S., Chen B., Song Y., Zhang T., Yang W., Shaman J. (2020). Substantial undocumented infection facilitates the rapid dissemination of novel coronavirus (SARS-CoV-2). Science.

[6] Shi J., Wen Z., Zhong G., Yang H., Wang C., Huang B., Liu R., He X., Shuai L., Sun Z. (2020). Susceptibility of ferrets, cats, dogs, and other domesticated animals to SARS–coronavirus 2. Science.

[7] Woo P.C.Y., Lau S.K.P., Lam C.S.F., Lau C.C.Y., Tsang A.K.L., Lau J.H.N., Bai R., Teng J.L.L., Tsang C.C.C., Wang M. (2012). Discovery of Seven Novel Mammalian and Avian Coronaviruses in the Genus Deltacoronavirus Supports Bat Coronaviruses as the Gene Source of Alphacoronavirus and Betacoronavirus and Avian Coronaviruses as the Gene Source of Gammacoronavirus and Deltacoronavirus. J. Virol..

[8] Lim Y.X., Ng Y.L., Tam J.P., Liu D.X. (2016). Human Coronaviruses: A Review of Virus–Host Interactions. Diseases.

[9] Perlman S., Netland J. (2009). Coronaviruses post-SARS: Update on replication and pathogenesis. Nat. Rev. Genet..

[10] Hu B., Ge X., Wang L.-F., Shi Z. (2015). Bat origin of human coronaviruses. Virol. J..

[11] Wang Q., Vlasova A.N., Kenney S.P., Saif L.J. (2019). Emerging and re-emerging coronaviruses in pigs. Curr. Opin. Virol..

[12] Cui J., Li F., Shi Z.-L. (2019). Origin and evolution of pathogenic coronaviruses. Nat. Rev. Genet..

[13] Yang Y.-L., Yu J.-Q., Huang Y.-W. (2020). Swine enteric alphacoronavirus (swine acute diarrhea syndrome coronavirus): An update three years after its discovery. Virus Res..

[14] Legnardi M., Tucciarone C.M., Franzo G., Cecchinato M. (2020). Infectious Bronchitis Virus Evolution, Diagnosis and Control. Vet. Sci..

[15] Zhang J. (2016). Porcine deltacoronavirus: Overview of infection dynamics, diagnostic methods, prevalence and genetic evolution. Virus Res..

[16] Wille M., Holmes E.C. (2020). Wild birds as reservoirs for diverse and abundant gamma- and deltacoronaviruses. FEMS Microbiol. Rev..

[17] Jung K., Hu H., Saif L.J. (2017). Calves are susceptible to infection with the newly emerged porcine deltacoronavirus, but not with the swine enteric alphacoronavirus, porcine epidemic diarrhea virus. Arch. Virol..

[18] Liang Q., Zhang H., Li B., Ding Q., Wang Y., Gao W., Guo D., Wei Z., Hu H. (2019). Susceptibility of Chickens to Porcine Deltacoronavirus Infection. Viruses.

[19] Boley P.A., Alhamo M.A., Lossie G., Yadav K.K., Vasquez-Lee M., Saif L.J., Kenney S.P., Boley P. (2020). Porcine Deltacoronavirus Infection and Transmission in Poultry, United States1. Emerg. Infect. Dis..

[20] Lednicky J.A., Tagliamonte M.S., White S.K., Elbadry M.A., Alam M.M., Stephenson C.J., Bonny T.S., Loeb J.C., Telisma T., Chavannes S. (2021). Emergence of porcine delta-coronavirus pathogenic infections among children in Haiti through independent zoonoses and convergent evolution. medRxiv.

[21] Li W., Hulswit R.J.G., Kenney S.P., Widjaja I., Jung K., Alhamo M.A., van Dieren B., van Kuppeveld F.J.M., Saif L.J., Bosch B.-J. (2018). Broad receptor engagement of an emerging global coronavirus may potentiate its diverse cross-species transmissibility. Proc. Natl. Acad. Sci. USA.

[22] Liu D.X., Fung T.S., Chong K.K., Shukla A., Hilgenfeld R. (2014). Accessory proteins of SARS-CoV and other coronaviruses. Antiviral Res..

[23] Michel C.J., Mayer C., Poch O., Thompson J.D. (2020). Characterization of accessory genes in coronavirus genomes. Virol. J..

[24] Fang P., Fang L., Hong Y., Liu X., Dong N., Ma P., Bi J., Wang D., Xiao S. (2017). Discovery of a novel accessory protein NS7a encoded by porcine deltacoronavirus. J. Gen. Virol..

[25] Fang P., Fang L., Liu X., Hong Y., Wang Y., Dong N., Ma P., Bi J., Wang D., Xiao S. (2016). Identification and subcellular localization of porcine deltacoronavirus accessory protein NS6. Virology.

[26] McBride R., Fielding B.C. (2012). The Role of Severe Acute Respiratory Syndrome (SARS)-Coronavirus Accessory Proteins in Virus Pathogenesis. Viruses.

[27] Tan Y.-J., Lim S.G., Hong W. (2006). Understanding the accessory viral proteins unique to the severe acute respiratory syndrome (SARS) coronavirus. Antivir. Res..

[28] Brisse M., Ly H. (2019). Comparative Structure and Function Analysis of the RIG-I-Like Receptors: RIG-I and MDA5. Front. Immunol..

[29] Streicher F., Jouvenet N. (2019). Stimulation of Innate Immunity by Host and Viral RNAs. Trends Immunol..

[30] Kawai T., Takahashi K., Sato S., Coban C., Kumar H., Kato H., Ishii K.J., Takeuchi O., Akira S. (2005). IPS-1, an adaptor triggering RIG-I- and Mda5-mediated type I interferon induction. Nat. Immunol..

[31] Takaoka A., Yanai H. (2006). Interferon signalling network in innate defence. Cell. Microbiol..

[32] Chan R.W., Chan M.C., Agnihothram S., Chan L.L., Kuok D.I., Fong J.H., Guan Y., Poon L.L., Baric R.S., Nicholls J.M. (2013). Tropism of and innate immune responses to the novel human betacoronavirus lineage C virus in human ex vivo respiratory organ cultures. J. Virol..

[33] Luo J., Fang L., Dong N., Fang P., . Ding Z., Wang D., Chen H., Xiao S. (2016). Porcine deltacoronavirus (PDCoV) infection suppresses RIG-I-mediated interferon-beta production. Virology.

[34] Roth-Cross J.K., Martínez-Sobrido L., Scott E.P., García-Sastre A., Weiss S.R. (2007). Inhibition of the Alpha/Beta Interferon Response by Mouse Hepatitis Virus at Multiple Levels. J. Virol..

[35] Blanco-Melo D., Nilsson-Payant B.E., Liu W.C., Uhl S., Hoagland D., Moller R., Jordan T.X., Oishi K., Panis M., Sachs D. (2020). Imbalanced Host Response to SARS-CoV-2 Drives Development of COVID-19. Cell.

[36] Sen G.C. (2001). Viruses and Interferons. Annu. Rev. Microbiol..

[37] Zinzula L., Tramontano E. (2013). Strategies of highly pathogenic RNA viruses to block dsRNA detection by RIG-I-like receptors: Hide, mask, hit. Antivir. Res..

[38] Niemeyer D., Zillinger T., Muth D., Zielecki F., Horvath G., Suliman T., Barchet W., Weber F., Drosten C., Müller M.A. (2013). Middle East Respiratory Syndrome Coronavirus Accessory Protein 4a Is a Type I Interferon Antagonist. J. Virol..

[39] Siu K.-L., Yeung M.L., Kok K.-H., Yuen K.-Y., Kew C., Lui P.-Y., Chan C.-P., Tse H., Woo P.C.Y., Jin D.-Y. (2014). Middle East Respiratory Syndrome Coronavirus 4a Protein Is a Double-Stranded RNA-Binding Protein That Suppresses PACT-Induced Activation of RIG-I and MDA5 in the Innate Antiviral Response. J. Virol..

[40] Fang P., Fang L., Ren J., Hong Y., Liu X., Zhao Y., Wang D., Peng G., Xiao S. (2018). Porcine Deltacoronavirus Accessory Protein NS6 Antagonizes Interferon Beta Production by Interfering with the Binding of RIG-I/MDA5 to Double-Stranded RNA. J. Virol..

[41] Lee J.Y., Kim S.-J., Myoung J. (2019). Middle East Respiratory Syndrome Coronavirus-Encoded ORF8b Inhibits RIG-I-Like Receptors in a Differential Mechanism. J. Microbiol. Biotechnol..

[42] Mibayashi M., Martínez-Sobrido L., Loo Y.-M., Caárdenas W.B., Gale M., García-Sastre A. (2007). Inhibition of Retinoic Acid-Inducible Gene I-Mediated Induction of Beta Interferon by the NS1 Protein of Influenza A Virus. J. Virol..

[43] Wang X., Li Y., Mao A., Li C., Li Y., Tien P. (2010). Hepatitis B virus X protein suppresses virus-triggered IRF3 activation and IFN-beta induction by disrupting the VISA-associated complex. Cell Mol. Immunol..

[44] Kopecky-Bromberg S.A., Martínez-Sobrido L., Frieman M., Baric R.A., Palese P. (2007). Severe Acute Respiratory Syndrome Coronavirus Open Reading Frame (ORF) 3b, ORF 6, and Nucleocapsid Proteins Function as Interferon Antagonists. J. Virol..

[45] Freundt E.C., Yu L., Park E., Lenardo M.J., Xu X.-N. (2009). Molecular Determinants for Subcellular Localization of the Severe Acute Respiratory Syndrome Coronavirus Open Reading Frame 3b Protein. J. Virol..

[46] Shi C.-S., Qi H.-Y., Boularan C., Huang N.-N., Abu-Asab M., Shelhamer J.H., Kehrl J.H. (2014). SARS-Coronavirus Open Reading Frame-9b Suppresses Innate Immunity by Targeting Mitochondria and the MAVS/TRAF3/TRAF6 Signalosome. J. Immunol..

[47] Gordon D.E., Jang G.M., Bouhaddou M., Xu J., Obernier K., White K.M., O’Meara M.J., Rezelj V.V., Guo J.Z., Swaney D.L. (2020). A SARS-CoV-2 protein interaction map reveals targets for drug repurposing. Nature.

[48] Wu J., Shi Y., Pan X., Wu S., Hou R., Zhang Y., Zhong T., Tang H., Du W., Wang L. (2021). SARS-CoV-2 ORF9b inhibits RIG-I-MAVS antiviral signaling by interrupting K63-linked ubiquitination of NEMO. Cell Rep..

[49] Yang Y., Ye F., Zhu N., Wang W., Deng Y., Zhao Z., Tan W. (2015). Middle East respiratory syndrome coronavirus ORF4b protein inhibits type I interferon production through both cytoplasmic and nuclear targets. Sci. Rep..

[50] Wong L.R., Ye Z.W., Lui P.Y., Zheng X., Yuan S., Zhu L., Fung S.Y., Yuen K.S., Siu K.L., Yeung M.L. (2020). Middle East Respiratory Syndrome Coronavirus ORF8b Accessory Protein Suppresses Type I IFN Expression by Impeding HSP70-Dependent Activation of IRF3 Kinase IKKepsilon. J. Immunol..

[51] Fang P., Fang L., Xia S., Ren J., Zhang J., Bai D., Zhou Y., Peng G., Zhao S., Xiao S. (2020). Porcine Deltacoronavirus Accessory Protein NS7a Antagonizes IFN-beta Production by Competing With TRAF3 and IRF3 for Binding to IKKepsilon. Front. Cell. Infect. Microbiol..

[52] Wong H.H., Fung T.S., Fang S., Huang M., Le M.T., Liu D.X. (2018). Accessory proteins 8b and 8ab of severe acute respiratory syndrome coronavirus suppress the interferon signaling pathway by mediating ubiquitin-dependent rapid degradation of interferon regulatory factor 3. Virology.

[53] Canton J., Fehr A.R., Fernandez-Delgado R., Gutierrez-Alvarez F.J., Sanchez-Aparicio M.T., Garcia-Sastre A., Perlman S., Enjuanes L., Sola I. (2018). MERS-CoV 4b protein interferes with the NF-kappaB-dependent innate immune response during infection. PLoS Pathog..

[54] Beidas M., Chehadeh W. (2018). Effect of Human Coronavirus OC43 Structural and Accessory Proteins on the Transcriptional Activation of Antiviral Response Elements. Intervirology.

[55] Yang Y., Zhang L., Geng H., Deng Y., Huang B., Guo Y., Zhao Z., Tan W. (2013). The structural and accessory proteins M, ORF 4a, ORF 4b, and ORF 5 of Middle East respiratory syndrome coronavirus (MERS-CoV) are potent interferon antagonists. Protein Cell.

[56] Li J.Y., Liao C.H., Wang Q., Tan Y.J., Luo R., Qiu Y., Ge X.Y. (2020). The ORF6, ORF8 and nucleocapsid proteins of SARS-CoV-2 inhibit type I interferon signaling pathway. Virus Res..

[57] Lei X., Dong X., Ma R., Wang W., Xiao X., Tian Z., Wang C., Wang Y., Li L., Ren L. (2020). Activation and evasion of type I interferon responses by SARS-CoV-2. Nat. Commun..

[58] Konno Y., Kimura I., Uriu K., Fukushi M., Irie T., Koyanagi Y., Sauter D., Gifford R.J., Consortium U.-C., Nakagawa S. (2020). SARS-CoV-2 ORF3b Is a Potent Interferon Antagonist Whose Activity Is Increased by a Naturally Occurring Elongation Variant. Cell Rep..

[59] Flower T.G., Buffalo C.Z., Hooy R.M., Allaire M., Ren X., Hurley J.H. (2021). Structure of SARS-CoV-2 ORF8, a rapidly evolving immune evasion protein. Proc. Natl. Acad. Sci. USA.

[60] Minakshi R., Padhan K., Rani M., Khan N., Ahmad F., Jameel S. (2009). The SARS Coronavirus 3a Protein Causes Endoplasmic Reticulum Stress and Induces Ligand-Independent Downregulation of the Type 1 Interferon Receptor. PLoS ONE.

[61] Frieman M., Yount B., Heise M., Kopecky-Bromberg S.A., Palese P., Baric R.S. (2007). Severe Acute Respiratory Syndrome Coronavirus ORF6 Antagonizes STAT1 Function by Sequestering Nuclear Import Factors on the Rough Endoplasmic Reticulum/Golgi Membrane. J. Virol..

[62] Cheng W., Chen S., Li R., Chen Y., Wang M., Guo D. (2015). Severe acute respiratory syndrome coronavirus protein 6 mediates ubiquitin-dependent proteosomal degradation of N-Myc (and STAT) interactor. Virol. Sin..

[63] Miorin L., Kehrer T., Sanchez-Aparicio M.T., Zhang K., Cohen P., Patel R.S., Cupic A., Makio T., Mei M., Moreno E. (2020). SARS-CoV-2 Orf6 hijacks Nup98 to block STAT nuclear import and antagonize interferon signaling. Proc. Nat. Acad. Sci. USA.

[64] Dedeurwaerder A., Olyslaegers D.A.J., Desmarets L.M.B., Roukaerts I.D.M., Theuns S., Nauwynck H.J. (2014). ORF7-encoded accessory protein 7a of feline infectious peritonitis virus as a counteragent against IFN-α-induced antiviral response. J. Gen. Virol..

[65] Koetzner C.A., Kuo L., Goebel S.J., Dean A.B., Parker M.M., Masters P.S. (2010). Accessory Protein 5a Is a Major Antagonist of the Antiviral Action of Interferon against Murine Coronavirus. J. Virol..

[66] Kint J., Dickhout A., Kutter J., Maier H.J., Britton P., Koumans J., Pijlman G.P., Fros J., Wiegertjes G., Forlenza M. (2015). Infectious Bronchitis Coronavirus Inhibits STAT1 Signaling and Requires Accessory Proteins for Resistance to Type I Interferon Activity. J. Virol..

[67] Shang J., Han N., Chen Z., Peng Y., Li L., Zhou H., Ji C., Meng J., Jiang T., Wu A. (2021). Compositional diversity and evolutionary pattern of coronavirus accessory proteins. Briefings Bioinform..

[68] Cruz J.L.G., Sola I., Becares M., Alberca B., Plana J., Enjuanes L., Zuñiga S. (2011). Coronavirus Gene 7 Counteracts Host Defenses and Modulates Virus Virulence. PLoS Pathog..

[69] Rabouw H.H., Langereis M.A., Knaap R.C.M., Dalebout T.J., Canton J., Sola I., Enjuanes L., Bredenbeek P.J., Kikkert M., De Groot R.J. (2016). Middle East Respiratory Coronavirus Accessory Protein 4a Inhibits PKR-Mediated Antiviral Stress Responses. PLoS Pathog..

[70] Nakagawa K., Narayanan K., Wada M., Makino S. (2018). Inhibition of Stress Granule Formation by Middle East Respiratory Syndrome Coronavirus 4a Accessory Protein Facilitates Viral Translation, Leading to Efficient Virus Replication. J. Virol..

[71] Zhao L., Jha B.K., Wu A., Elliott R., Ziebuhr J., Gorbalenya A., Silverman R.H., Weiss S.R. (2012). Antagonism of the Interferon-Induced OAS-RNase L Pathway by Murine Coronavirus ns2 Protein Is Required for Virus Replication and Liver Pathology. Cell Host Microbe.

[72] Goldstein S.A., Thornbrough J.M., Zhang R., Jha B.K., Li Y., Elliott R., Quiroz-Figueroa K., Chen A.I., Silverman R.H., Weiss S.R. (2017). Lineage A Betacoronavirus NS2 Proteins and the Homologous Torovirus Berne pp1a Carboxy-Terminal Domain Are Phosphodiesterases That Antagonize Activation of RNase L. J. Virol..

[73] Thornbrough J.M., Jha B.K., Yount B., Goldstein S.A., Li Y., Elliott R., Sims A.C., Baric R.S., Silverman R.H., Weiss S.R. (2016). Middle East Respiratory Syndrome Coronavirus NS4b Protein Inhibits Host RNase L Activation. mBio.

[74] Zhou F., Yu T., Du R., Fan G., Liu Y., Liu Z., Xiang J., Wang Y., Song B., Gu X. (2020). Clinical course and risk factors for mortality of adult inpatients with COVID-19 in Wuhan, China: A retrospective cohort study. Lancet.

[75] Freeman T.L., Swartz T.H. (2020). Targeting the NLRP3 Inflammasome in Severe COVID-19. Front. Immunol..

[76] Sendler M., Brandt C.V.D., Glaubitz J., Wilden A., Golchert J., Weiss F.U., Homuth G., Chama L.L.D.F., Mishra N., Mahajan U.M. (2020). NLRP3 Inflammasome Regulates Development of Systemic Inflammatory Response and Compensatory Anti-Inflammatory Response Syndromes in Mice With Acute Pancreatitis. Gastroenterology.

[77] Martinon F., Burns K., Tschopp J. (2002). The inflammasome: A molecular platform triggering activation of inflammatory caspases and processing of proIL-beta. Mol. Cell.

[78] Siu K., Yuen K., Castano-Rodriguez C., Ye Z., Yeung M., Fung S., Yuan S., Chan C., Yuen K., Enjuanes L. (2019). Severe acute respiratory syndrome Coronavirus ORF3a protein activates the NLRP3 inflammasome by promoting TRAF3-dependent ubiquitination of ASC. FASEB J..

[79] Shi C.-S., Nabar N.R., Huang N.-N., Kehrl J.H. (2019). SARS-Coronavirus Open Reading Frame-8b triggers intracellular stress pathways and activates NLRP3 inflammasomes. Cell Death Discov..

[80] Paniri A., Akhavan-Niaki H. (2020). Emerging role of IL-6 and NLRP3 inflammasome as potential therapeutic targets to combat COVID-19: Role of lncRNAs in cytokine storm modulation. Life Sci..

[81] Kanzawa N., Nishigaki K., Hayashi T., Ishii Y., Furukawa S., Niiro A., Yasui F., Kohara M., Morita K., Matsushima K. (2006). Augmentation of chemokine production by severe acute respiratory syndrome coronavirus 3a/X1 and 7a/X4 proteins through NF-kappaB activation. FEBS Lett..

[82] Obitsu S., Ahmed N., Nishitsuji H., Hasegawa A., Nakahama K.-I., Morita I., Nishigaki K., Hayashi T., Masuda T., Kannagi M. (2009). Potential enhancement of osteoclastogenesis by severe acute respiratory syndrome coronavirus 3a/X1 protein. Arch. Virol..

[83] Kopecky-Bromberg S.A., Martinez-Sobrido L., Palese P. (2006). 7a Protein of Severe Acute Respiratory Syndrome Coronavirus Inhibits Cellular Protein Synthesis and Activates p38 Mitogen-Activated Protein Kinase. J. Virol..

[84] Varshney B., Agnihothram S., Tan Y.J., Baric R., Lal S.K. (2012). SARS coronavirus 3b accessory protein modulates transcriptional activity of RUNX1b. PLoS ONE.

[85] Varshney B., Lal S.K. (2011). SARS-CoV accessory protein 3b induces AP-1 transcriptional activity through activation of JNK and ERK pathways. Biochemistry.

[86] Ding Z., An K., Xie L., Wu W., Zhang R., Wang D., Fang Y., Chen H., Xiao S., Fang L. (2017). Transmissible gastroenteritis virus infection induces NF-kappaB activation through RLR-mediated signaling. Virology.

[87] Jung K., Miyazaki A., Hu H., Saif L.J. (2018). Susceptibility of porcine IPEC-J2 intestinal epithelial cells to infection with porcine deltacoronavirus (PDCoV) and serum cytokine responses of gnotobiotic pigs to acute infection with IPEC-J2 cell culture-passaged PDCoV. Vet. Microbiol..

[88] Kaewborisuth C., Koonpaew S., Srisutthisamphan K., Viriyakitkosol R., Jaru-Ampornpan P., Jongkaewwattana A. (2020). PEDV ORF3 Independently Regulates IkappaB Kinase beta-Mediated NF-kappaB and IFN-beta Promoter Activities. Pathogens.

[89] Wu Z., Cheng L., Xu J., Li P., Li X., Zou D., Zhang Y., Wang X., Wu X., Shen Y. (2020). The accessory protein ORF3 of porcine epidemic diarrhea virus inhibits cellular interleukin-6 and interleukin-8 productions by blocking the nuclear factor-kappaB p65 activation. Vet. Microbiol..

[90] Cruz J.L., Becares M., Sola I., Oliveros J.C., Enjuanes L., Zuniga S. (2013). Alphacoronavirus protein 7 modulates host innate immune response. J. Virol..

[91] Fung T.S., Liu D.X. (2014). Coronavirus infection, ER stress, apoptosis and innate immunity. Front. Microbiol..

[92] Oakes S.A., Papa F.R. (2015). The Role of Endoplasmic Reticulum Stress in Human Pathology. Annu. Rev. Pathol. Mech. Dis..

[93] DeDiego M.L., Nieto-Torres J.L., Guardeno J.M.J., Regla-Nava J.A., Álvarez E., Oliveros J.C., Zhao J., Fett C., Perlman S., Enjuanes L. (2011). Severe Acute Respiratory Syndrome Coronavirus Envelope Protein Regulates Cell Stress Response and Apoptosis. PLoS Pathog..

[94] Bechill J., Chen Z., Brewer J.W., Baker S.C. (2008). Coronavirus Infection Modulates the Unfolded Protein Response and Mediates Sustained Translational Repression. J. Virol..

[95] Xue M., Fu F., Ma Y., Zhang X., Li L., Feng L., Liu P. (2018). The PERK Arm of the Unfolded Protein Response Negatively Regulates Transmissible Gastroenteritis Virus Replication by Suppressing Protein Translation and Promoting Type I Interferon Production. J. Virol..

[96] Liao Y., Fung T.S., Huang M., Fang S.G., Zhong Y., Liu D.X. (2013). Upregulation of CHOP/GADD153 during Coronavirus Infectious Bronchitis Virus Infection Modulates Apoptosis by Restricting Activation of the Extracellular Signal-Regulated Kinase Pathway. J. Virol..

[97] Banerjee A., Czinn S.J., Reiter R.J., Blanchard T.G. (2020). Crosstalk between endoplasmic reticulum stress and anti-viral activities: A novel therapeutic target for COVID-19. Life Sci..

[98] Köseler A., Sabirli R., Gören T., Türkçüer I., Kurt Ö. (2020). Endoplasmic Reticulum Stress Markers in SARS-COV-2 Infection and Pneumonia: Case-Control Study. In Vivo.

[99] Ding L., Li J., Li W., Fang Z., Li N., Wu S., Li J., Hong M. (2018). p53- and ROS-mediated AIF pathway involved in TGEV-induced apoptosis. J. Vet. Med. Sci..

[100] Kim Y., Lee C. (2014). Porcine epidemic diarrhea virus induces caspase-independent apoptosis through activation of mitochondrial apoptosis-inducing factor. Virology.

[101] Xu X., Xu Y., Zhang Q., Yang F., Yin Z., Wang L., Li Q. (2019). Porcine epidemic diarrhea virus infections induce apoptosis in Vero cells via a reactive oxygen species (ROS)/p53, but not p38 MAPK and SAPK/JNK signalling pathways. Vet. Microbiol..

[102] Shuid A.N., Safi N., Haghani A., Mehrbod P., Haron M.S.R., Tan S.W., Omar A.R. (2015). Apoptosis transcriptional mechanism of feline infectious peritonitis virus infected cells. Apoptosis.

[103] Meessen-Pinard M., Le Coupanec A., Desforges M., Talbot P.J. (2017). Pivotal Role of Receptor-Interacting Protein Kinase 1 and Mixed Lineage Kinase Domain-Like in Neuronal Cell Death Induced by the Human Neuroinvasive Coronavirus OC43. J. Virol..

[104] Collins A.R. (2002). In Vitro Detection of Apoptosis in Monocytes/Macrophages Infected with Human Coronavirus. Clin. Vaccine Immunol..

[105] Yan H., Xiao G., Zhang J., Hu Y., Yuan F., Cole D., Zheng C., Gao G.F. (2004). SARS coronavirus induces apoptosis in Vero E6 Cells. J. Med. Virol..

[106] Yeung M.-L., Yao Y., Jia L., Chan J.F.W., Chan K.-H., Cheung K.-F., Chen H., Poon V.K.M., Tsang A.K.L., To K.K. (2016). MERS coronavirus induces apoptosis in kidney and lung by upregulating Smad7 and FGF2. Nat. Microbiol..

[107] Chu H., Zhou J., Wong B.H.-Y., Li C., Chan J.F.-W., Cheng Z.-S., Yang D., Wang D., Lee A.C.Y., Li C. (2016). Middle East Respiratory Syndrome Coronavirus Efficiently Infects Human Primary T Lymphocytes and Activates the Extrinsic and Intrinsic Apoptosis Pathways. J. Infect. Dis..

[108] Chen C.-J., Makino S. (2002). Murine Coronavirus-Induced Apoptosis in 17Cl-1 Cells Involves a Mitochondria-Mediated Pathway and Its Downstream Caspase-8 Activation and Bid Cleavage. Virology.

[109] Liu Y., Zhang X. (2007). Murine coronavirus-induced oligodendrocyte apoptosis is mediated through the activation of the Fas signaling pathway. Virology.

[110] Han X., Tian Y., Guan R., Gao W., Yang X., Zhou L., Wang H. (2017). Infectious Bronchitis Virus Infection Induces Apoptosis during Replication in Chicken Macrophage HD11 Cells. Viruses.

[111] Lee Y.J., Lee C. (2018). Porcine deltacoronavirus induces caspase-dependent apoptosis through activation of the cytochrome c -mediated intrinsic mitochondrial pathway. Virus Res..

[112] Jung K., Hu H., Saif L.J. (2016). Porcine deltacoronavirus induces apoptosis in swine testicular and LLC porcine kidney cell lines in vitro but not in infected intestinal enterocytes in vivo. Vet. Microbiol..

[113] Cottam E.M., Maier H.J., Manifava M., Vaux L.C., Chandra-Schoenfelder P., Gerner W., Britton P., Ktistakis N.T., Wileman T. (2011). Coronavirus nsp6 proteins generate autophagosomes from the endoplasmic reticulum via an omegasome intermediate. Autophagy.

[114] Qin P., Du E.-Z., Luo W.-T., Yang Y.-L., Zhang Y.-Q., Wang B., Huang Y.-W. (2019). Characteristics of the Life Cycle of Porcine Deltacoronavirus (PDCoV) In Vitro: Replication Kinetics, Cellular Ultrastructure and Virion Morphology, and Evidence of Inducing Autophagy. Viruses.

[115] García-Pérez B.E., González-Rojas J.A., Salazar M.I., Torres-Torres C., Castrejón-Jiménez N.S. (2020). Taming the Autophagy as a Strategy for Treating COVID-19. Cells.

[116] Domdom M.-A., Brest P., Grosjean I., Roméo B., Landi M.T., Gal J., Klionsky D.J., Hofman P., Mograbi B. (2020). A multifactorial score including autophagy for prognosis and care of COVID-19 patients. Autophagy.

[117] Gorshkov K., Chen C.Z., Bostwick R., Rasmussen L., Xu M., Pradhan M., Tran B.N., Zhu W., Shamim K., Huang W. (2020). The SARS-CoV-2 cytopathic effect is blocked with autophagy modulators. bioRxiv.

[118] Sung S.C., Chao C.Y., Jeng K.S., Yang J.Y., Lai M.M. (2009). The 8ab protein of SARS-CoV is a luminal ER membrane-associated protein and induces the activation of ATF6. Virology.

[119] Ye Z., Wong C.K., Li P., Xie Y. (2008). A SARS-CoV protein, ORF-6, induces caspase-3 mediated, ER stress and JNK-dependent apoptosis. Biochim. Biophys. Acta (BBA) Gen. Subj..

[120] Padhan K., Minakshi R., Bin Towheed M.A., Jameel S. (2008). Severe acute respiratory syndrome coronavirus 3a protein activates the mitochondrial death pathway through p38 MAP kinase activation. J. Gen. Virol..

[121] Yue Y., Nabar N.R., Shi C.-S., Kamenyeva O., Xiao X., Hwang I.-Y., Wang M., Kehrl J.H. (2018). SARS-Coronavirus Open Reading Frame-3a drives multimodal necrotic cell death. Cell Death Dis..

[122] Tan Y.X., Tan T.H., Lee M.J., Tham P.Y., Gunalan V., Druce J., Birch C., Catton M., Fu N.Y., Yu V.C. (2007). Induction of apoptosis by the severe acute respiratory syndrome coronavirus 7a protein is dependent on its interaction with the Bcl-XL protein. J. Virol..

[123] Tan Y.-J., Fielding B.C., Goh P.-Y., Shen S., Tan T.H.P., Lim S.G., Hong W. (2004). Overexpression of 7a, a Protein Specifically Encoded by the Severe Acute Respiratory Syndrome Coronavirus, Induces Apoptosis via a Caspase-Dependent Pathway. J. Virol..

[124] Chen C.-Y., Ping Y.-H., Lee H.-C., Chen K.-H., Lee Y.-M., Chan Y.-J., Lien T.-C., Jap T.-S., Lin C.-H., Kao L.-S. (2007). Open Reading Frame 8a of the Human Severe Acute Respiratory Syndrome Coronavirus Not Only Promotes Viral Replication but Also Induces Apoptosis. J. Infect. Dis..

[125] Sharma K., Åkerström S., Sharma A.K., Chow V., Teow S., Abrenica B., Booth S.A., Booth T.F., Mirazimi A., Lal S.K. (2011). SARS-CoV 9b Protein Diffuses into Nucleus, Undergoes Active Crm1 Mediated Nucleocytoplasmic Export and Triggers Apoptosis When Retained in the Nucleus. PLoS ONE.

[126] Ren Y., Shu T., Wu D., Mu J., Wang C., Huang M., Han Y., Zhang X.Y., Zhou W., Qiu Y. (2020). The ORF3a protein of SARS-CoV-2 induces apoptosis in cells. Cell Mol. Immunol..

[127] Si F., Hu X., Wang C., Chen B., Wang R., Dong S., Yu R., Li Z. (2020). Porcine Epidemic Diarrhea Virus (PEDV) ORF3 Enhances Viral Proliferation by Inhibiting Apoptosis of Infected Cells. Viruses.

[128] Fung T.S., Liu D.X. (2019). The ER stress sensor IRE1 and MAP kinase ERK modulate autophagy induction in cells infected with coronavirus infectious bronchitis virus. Virology.

[129] Fung T.S., Liao Y., Liu D.X. (2014). The Endoplasmic Reticulum Stress Sensor IRE1 Protects Cells from Apoptosis Induced by the Coronavirus Infectious Bronchitis Virus. J. Virol..

[130] Zou D., Xu J., Duan X., Xu X., Li P., Cheng L., Zheng L., Li X., Zhang Y., Wang X. (2019). Porcine epidemic diarrhea virus ORF3 protein causes endoplasmic reticulum stress to facilitate autophagy. Vet. Microbiol..

[131] Nieva J.L., Madan V., Carrasco L. (2012). Viroporins: Structure and biological functions. Nat. Rev. Genet..

[132] De Jong A.S., Visch H.J., de Mattia F., van Dommelen M.M., Swarts H.G., Luyten T., Callewaert G., Melchers W.J., Willems P.H., van Kuppeveld F.J. (2006). The coxsackievirus 2B protein increases efflux of ions from the endoplasmic reticulum and Golgi, thereby inhibiting protein trafficking through the Golgi. J Biol. Chem..

[133] Pinto L.H., Holsinger L.J., Lamb R.A. (1992). Influenza virus M2 protein has ion channel activity. Cell.

[134] Pavlovic D., Neville D.C.A., Argaud O., Blumberg B., Dwek R.A., Fischer W.B., Zitzmann N. (2003). The hepatitis C virus p7 protein forms an ion channel that is inhibited by long-alkyl-chain iminosugar derivatives. Proc. Natl. Acad. Sci. USA.

[135] Wilson L., Mckinlay C., Gage P., Ewart G. (2004). SARS coronavirus E protein forms cation-selective ion channels. Virology.

[136] Surya W., Li Y., Verdià-Bàguena C., Aguilella V.M., Torres J. (2015). MERS coronavirus envelope protein has a single transmembrane domain that forms pentameric ion channels. Virus Res..

[137] Singh Tomar P.P., Arkin I.T. (2020). SARS-CoV-2 E protein is a potential ion channel that can be inhibited by Gliclazide and Memantine. Biochem. Biophys. Res. Commun..

[138] Ye Y., Hogue B.G. (2007). Role of the Coronavirus E Viroporin Protein Transmembrane Domain in Virus Assembly. J. Virol..

[139] Wang K., Xie S., Sun B. (2011). Viral proteins function as ion channels. Biochim. Biophys. Acta (BBA) Biomembr..

[140] Chien T.H., Chiang Y.L., Chen C.P., Henklein P., Hanel K., Hwang I.S., Willbold D., Fischer W.B. (2013). Assembling an ion channel: ORF 3a from SARS-CoV. Biopolymers.

[141] Chen C.C., Kruger J., Sramala I., Hsu H.J., Henklein P., Chen Y.M., Fischer W.B. (2011). ORF8a of SARS-CoV forms an ion channel: Experiments and molecular dynamics simulations. Biochim. Biophys. Acta.

[142] Wang K., Lu W., Chen J., Xie S., Shi H., Hsu H., Yu W., Xu K., Bian C., Fischer W.B. (2012). PEDV ORF3 encodes an ion channel protein and regulates virus production. FEBS Lett..

[143] Zhang R., Wang K., Lv W., Yu W., Xie S., Xu K., Schwarz W., Xiong S., Sun B. (2014). The ORF4a protein of human coronavirus 229E functions as a viroporin that regulates viral production. Biochim. Biophys. Acta (BBA) Biomembr..

[144] Zhang R., Wang K., Ping X., Yu W., Qian Z., Xiong S., Sun B. (2015). The ns12.9 Accessory Protein of Human Coronavirus OC43 Is a Viroporin Involved in Virion Morphogenesis and Pathogenesis. J. Virol..

[145] Almazán F., DeDiego M.L., Sola I., Zuñiga S., Nieto-Torres J.L., Marquez-Jurado S., Andrés G., Enjuanes L. (2013). Engineering a Replication-Competent, Propagation-Defective Middle East Respiratory Syndrome Coronavirus as a Vaccine Candidate. mBio.

[146] DeDiego M.L., Álvare E., Almazán F., Rejas M.T., Lamirande E., Roberts A., Shieh W.-J., Zaki S.R., Subbarao K., Enjuanes L. (2006). A Severe Acute Respiratory Syndrome Coronavirus That Lacks the E Gene Is Attenuated In Vitro and In Vivo. J. Virol..

[147] Kuo L., Masters P.S. (2003). The Small Envelope Protein E Is Not Essential for Murine Coronavirus Replication. J. Virol..

[148] Lu W., Zheng B.-J., Xu K., Schwarz W., Du L., Wong C.K.L., Chen J., Duan S., Deubel V., Sun B. (2006). Severe acute respiratory syndrome-associated coronavirus 3a protein forms an ion channel and modulates virus release. Proc. Natl. Acad. Sci. USA.

[149] Chan C.-M., Tsoi H., Chan W.-M., Zhai S., Wong C.O., Yao X., Chan W.Y., Tsui S.K.-W., Chan H.Y.E. (2009). The ion channel activity of the SARS-coronavirus 3a protein is linked to its pro-apoptotic function. Int. J. Biochem. Cell Biol..

[150] Castaño-Rodriguez C., Honrubia J.M., Gutiérrez-Álvarez J., DeDiego M.L., Nieto-Torres J.L., Jimenez-Guardeño J.M., Regla-Nava J.A., Fernandez-Delgado R., Verdia-Báguena C., Queralt-Martín M. (2018). Role of Severe Acute Respiratory Syndrome Coronavirus Viroporins E, 3a, and 8a in Replication and Pathogenesis. mBio.

[151] Bai D., Fang L., Xia S., Ke W., Wang J., Wu X., Fang P., Xiao S. (2020). Porcine deltacoronavirus (PDCoV) modulates calcium influx to favor viral replication. Virology.

[152] Hyser J.M., Utama B., Crawford S.E., Broughman J.R., Estes M.K. (2013). Activation of the Endoplasmic Reticulum Calcium Sensor STIM1 and Store-Operated Calcium Entry by Rotavirus Requires NSP4 Viroporin Activity. J. Virol..

[153] Stouffer A.L., Acharya R., Salom D., Levine A.S., Di Costanzo L., Soto C.S., Tereshko V., Nanda V., Stayrook S., DeGrado W.F. (2008). Structural basis for the function and inhibition of an influenza virus proton channel. Nat. Cell Biol..

[154] Luik P., Chew C., Aittoniemi J., Chang J., Wentworth P., Dwek R.A., Biggin P.C., Venien-Bryan C., Zitzmann N. (2009). The 3-dimensional structure of a hepatitis C virus p7 ion channel by electron microscopy. Proc. Natl. Acad. Sci. USA.

[155] Xie S., Wang K., Yu W., Lu W., Xu K., Wang J., Ye B., Schwarz W., Jin Q., Sun B. (2011). DIDS blocks a chloride-dependent current that is mediated by the 2B protein of enterovirus 71. Cell Res..

[156] Griffin S.D.C. (2009). Plugging the holes in hepatitis C virus antiviral therapy. Proc. Natl. Acad. Sci. USA.

[157] Griffin S., Beales L.P., Clarke D.S., Worsfold O., Evans S., Jaeger J., Harris M.P., Rowlands D.J. (2003). The p7 protein of hepatitis C virus forms an ion channel that is blocked by the antiviral drug, Amantadine. FEBS Lett..

[158] Cady S., Schmidt-Rohr K., Wang J., Soto C.S., DeGrado W.F., Hong M. (2010). Structure of the amantadine binding site of influenza M2 proton channels in lipid bilayers. Nat. Cell Biol..

[159] Ewart G.D., Mills K., Cox G.B., Gage P.W. (2002). Amiloride derivatives block ion channel activity and enhancement of virus-like particle budding caused by HIV-1 protein Vpu. Eur. Biophys. J..

[160] Watanabe S., Watanabe T., Kawaoka Y. (2009). Influenza A Virus Lacking M2 Protein as a Live Attenuated Vaccine. J. Virol..

[161] Haijema B.J., Volders H., Rottier P.J.M. (2004). Live, Attenuated Coronavirus Vaccines through the Directed Deletion of Group-Specific Genes Provide Protection against Feline Infectious Peritonitis. J. Virol..

[162] Dedeurwaerder A., Desmarets L.M., Olyslaegers D.A., Vermeulen B.L., Dewerchin H.L., Nauwynck H.J. (2013). The role of accessory proteins in the replication of feline infectious peritonitis virus in peripheral blood monocytes. Vet. Microbiol..

[163] Sola I., Alonso S., Zúñiga S., Balasch M., Plana-Durán J., Enjuanes L. (2003). Engineering the Transmissible Gastroenteritis Virus Genome as an Expression Vector Inducing Lactogenic Immunity. J. Virol..

[164] Pewe L., Zhou H., Netland J., Tangudu C., Olivares H., Shi L., Look D., Gallagher T., Perlman S. (2005). A Severe Acute Respiratory Syndrome-Associated Coronavirus-Specific Protein Enhances Virulence of an Attenuated Murine Coronavirus. J. Virol..

[165] Menachery V.D., Mitchell H.D., Cockrell A.S., Gralinski L.E., Yount B.L., Graham R.L., McAnarney E.T., Douglas M.G., Scobey T., Beall A. (2017). MERS-CoV Accessory ORFs Play Key Role for Infection and Pathogenesis. mBio.

[166] De Haan C.A., Masters P.S., Shen X., Weiss S., Rottier P.J. (2002). The group-specific murine coronavirus genes are not essential, but their deletion, by reverse genetics, is attenuating in the natural host. Virology.

[167] Van Beurden S.J., Berends A.J., Kramer-Kuhl A., Spekreijse D., Chenard G., Philipp H.C., Mundt E., Rottier P.J.M., Verheije M.H. (2018). Recombinant live attenuated avian coronavirus vaccines with deletions in the accessory genes 3ab and/or 5ab protect against infectious bronchitis in chickens. Vaccine.

[168] Zhao X., Jiang Y., Cheng X., Yu Y., Gao M., Zhou S. (2019). Pathogenicity of a QX-like strain of infectious bronchitis virus and effects of accessory proteins 3a and 3b in chickens. Vet. Microbiol..

[169] Kint J., Fernandez-Gutierrez M., Maier H.J., Britton P., Langereis M.A., Koumans J., Wiegertjes G.F., Forlenza M. (2015). Activation of the Chicken Type I Interferon Response by Infectious Bronchitis Coronavirus. J. Virol..

[170] Zhang M., Li W., Zhou P., Liu D., Luo R., Jongkaewwattana A., He Q. (2019). Genetic manipulation of porcine deltacoronavirus reveals insights into NS6 and NS7 functions: A novel strategy for vaccine design. Emerg. Microbes Infect..

[171] Ye C., Chiem K., Park J.G., Oladunni F., Platt R.N., Anderson T., Almazan F., de la Torre J.C., Martinez-Sobrido L. (2020). Rescue of SARS-CoV-2 from a Single Bacterial Artificial Chromosome. mBio.

[172] Xie X., Muruato A., Lokugamage K.G., Narayanan K., Zhang X., Zou J., Liu J., Schindewolf C., Bopp N.E., Aguilar P.V. (2020). An Infectious cDNA Clone of SARS-CoV-2. Cell Host Microbe.

[173] Chiem K., Morales Vasquez D., Park J.G., Platt R.N., Anderson T., Walter M.R., Kobie J.J., Ye C., Martinez-Sobrido L. (2021). Generation and Characterization of recombinant SARS-CoV-2 expressing reporter genes. J. Virol..

[174] Xie X., Muruato A.E., Zhang X., Lokugamage K.G., Fontes-Garfias C.R., Zou J., Liu J., Ren P., Balakrishnan M., Cihlar T. (2020). A nanoluciferase SARS-CoV-2 for rapid neutralization testing and screening of anti-infective drugs for COVID-19. Nat. Commun..

[175] Silvas J., Morales-Vasquez D., Park J.G., Chiem K., Torrelles J., Platt R.N., Anderson T., Ye C., Martinez-Sobrido L. (2021). Contribution of SARS-CoV-2 accessory proteins to viral pathogenicity in K18 hACE2 transgenic mice. bioRxiv.

[176] Keep S., Oade M.S., Lidzbarski-Silvestre F., Bentley K., Stevenson-Leggett P., Freimanis G.L., Tennakoon C., Sanderson N., Hammond J.A., Jones R.C. (2020). Multiple novel non-canonically transcribed sub-genomic mRNAs produced by avian coronavirus infectious bronchitis virus. J. Gen. Virol..

[177] Finkel Y., Mizrahi O., Nachshon A., Weingarten-Gabbay S., Morgenstern D., Yahalom-Ronen Y., Tamir H., Achdout H., Stein D., Israeli O. (2021). The coding capacity of SARS-CoV-2. Nature.

[178] Nelson C.W., Ardern Z., Goldberg T.L., Meng C., Kuo C.H., Ludwig C., Kolokotronis S.O., Wei X. (2020). Dynamically evolving novel overlapping gene as a factor in the SARS-CoV-2 pandemic. Elife.

[179] Peng Q., Fang L., Ding Z., Wang D., Peng G., Xiao S. (2020). Rapid manipulation of the porcine epidemic diarrhea virus genome by CRISPR/Cas9 technology. J. Virol. Methods.

